# Secular trends of incidence and prevalence of Parkinsonism subtypes: a cohort study in the United Kingdom

**DOI:** 10.1093/eurpub/ckag031

**Published:** 2026-04-07

**Authors:** Xihang Chen, Nicola L Barclay, Marta Pineda-Moncusí, Martí Català, Laura Molina-Porcel, Wai Yi Man, Antonella Delmestri, Daniel Prieto-Alhambra, Annika M Jödicke, Danielle Newby

**Affiliations:** Nuffield Department of Orthopaedics, Rheumatology and Musculoskeletal Sciences, University of Oxford, Oxford, United Kingdom; Nuffield Department of Orthopaedics, Rheumatology and Musculoskeletal Sciences, University of Oxford, Oxford, United Kingdom; Nuffield Department of Orthopaedics, Rheumatology and Musculoskeletal Sciences, University of Oxford, Oxford, United Kingdom; Nuffield Department of Orthopaedics, Rheumatology and Musculoskeletal Sciences, University of Oxford, Oxford, United Kingdom; Neurological Tissue Bank of the Biobanc-Hospital Clinic-Fundació de Recerca Clínic Barcelona-Institut d’Investigacions Biomèdiques August Pi i Sunyer (FRCB-IDIBAPS), Barcelona, Spain; Alzheimer’s Disease and Other Cognitive Disorders Unit, Neurology Service, Hospital Clínic, FRCB-IDIBAPS, University of Barcelona, Barcelona, Spain; Nuffield Department of Orthopaedics, Rheumatology and Musculoskeletal Sciences, University of Oxford, Oxford, United Kingdom; Nuffield Department of Orthopaedics, Rheumatology and Musculoskeletal Sciences, University of Oxford, Oxford, United Kingdom; Nuffield Department of Orthopaedics, Rheumatology and Musculoskeletal Sciences, University of Oxford, Oxford, United Kingdom; Department of Medical Informatics, Erasmus University Medical Centre, Rotterdam, The Netherlands; Nuffield Department of Orthopaedics, Rheumatology and Musculoskeletal Sciences, University of Oxford, Oxford, United Kingdom; Nuffield Department of Orthopaedics, Rheumatology and Musculoskeletal Sciences, University of Oxford, Oxford, United Kingdom

## Abstract

To evaluate secular trends of incidence and prevalence of Parkinson’s Disease (PD), Vascular Parkinsonism (VP), and Drug-induced Parkinsonism from 2007 to 2021 in the UK. We used primary care data, Clinical Practice Research Datalink GOLD, from the UK. Individuals were included if they were registered from January 2007 to December 2021 with at least one year of prior observation. Age-standardized and crude incidence and prevalence were calculated annually; age-standardized rates were stratified by sex, and crude rates by age and sex. From 2007 to 2019, the age-standardized incidence of PD decreased from 35.61 (95% confidence interval: 33.97–37.30) to 31.27 (29.27–33.37) per 100 000 person-years. The prevalence of PD increased from 0.21% (0.21%–0.22%) in 2007, peaking in 2016 at 0.23% (0.23%–0.24%). The number of VP diagnoses has increased since 2010, whereas the incidence and prevalence of DIP remained stable. Incidence and prevalence increased with age and were generally higher in males, except for DIP, which was slightly higher in females. Crude rates showed similar trends. Though Parkinson Disease incidence has declined, prevalence has risen, suggesting improved survival. VP rates have increased, possibly due to improvements in diagnostic screening. Drug-induced Parkinsonism rates remained stable. With an aging UK population, Parkinsonism subtypes pose a growing burden.

## Introduction

Parkinsonism is an umbrella term used to describe a syndrome characterized by symptoms commonly associated with Parkinson’s disease (PD) including muscle rigidity, tremors and slowness of movement [1]. Parkinsonism can also be caused by other factors, with other subtypes including Vascular Parkinsonism (VP) and Drug-induced Parkinsonism (DIP) as well as other progressive brain conditions [1]. VP is caused by ischemic cerebrovascular diseases which affect the brain areas involved in movement control, such as the basal ganglia [2]. On the other hand, DIP is caused by medications that block dopamine receptors in the brain [3]. Drugs known to induce DIP include certain antipsychotics, antidepressants, and antiepileptics; however, symptoms can be reversible after the withdrawal [3].

Critically, there is a lack of up-to-date evidence on the secular trends of the incidence and prevalence of these Parkinsonism subtypes, particularly in different sex and age groups. Studies have examined the incidence and prevalence of PD; however, they have not accounted for other Parkinsonism subtypes, and temporal trends in PD vary globally [4–6]. For example, a study in Norway has shown stable incidence and increasing prevalence [4] while a study in South Korea has shown increasing incidence and prevalence [5]. Moreover, although other studies considered Parkinsonism and subtypes, they were conducted regionally which may affect its generalizability [7]. Furthermore, although these two studies stratified results by age and sex, they did not consider temporal trends within each group.

In the light of this, a comprehensive assessment of pattern of Parkinsonism diagnosis over time is vital to better inform decisions regarding screening and treatment. Therefore, the aim of this study is to examine the secular trends of incidence and prevalence of PD, VP, and DIP from 2007 to 2021 in the UK for the whole population and stratified by age and sex.

## Methods

### Study design and data sources

This was a descriptive, population-based cohort study using routinely collected primary care data from the UK. The database for this study was the Clinical Practice Research Datalink (CPRD) GOLD database. CPRD GOLD is an established database documenting the pseudo-anonymized patient-level data contributed by General Practitioners (GPs) across the UK. By January 2024, CPRD GOLD contains > 21.3 million current and historical patients, with nearly 3 million current patients, counting for about 4% of the UK population overall [8]. CPRD GOLD was mapped to Observational Medical Outcomes Partnership (OMOP) (Common Data Model) [9] to standardize analytics. The use of CPRD GOLD for this study was approved through CPRD’s Research Data Governance Process (Protocol number: 22_002351). Individual consent was not necessary, as the CPRD data for this study are de-identified and approved for research use by the UK Health Research Authority and the NHS Health and Social Care Research Ethics Committee.

### Study participants and time at risk

People were eligible for this study if they were aged above 18 with at least one year of prior observation registered in CPRD GOLD from 1 January 2007 to 31 December 2021. This formed the background population and was used to calculate time at risk in person-years. Individuals were classified as having PD, VP, or DIP based on the presence of at least one relevant diagnostic Systematized Nomenclature of Medicine Clinical Terms (SNOMED CT) code recorded in primary care during study period; no requirement for repeated codes, symptom codes or treatment prescriptions was applied in the definition. Due to the chronic nature of PD or VP, individuals diagnosed with PD or VP were considered to have the condition from the date of their first outcome of interest within the study period until whichever occurred first: the practice stopped contributing to the database, the patient left the practice, the date of death, or 31 December 2021. In contrast, because DIP is not chronic in nature, any record was considered to last only 1 year.

### Outcome definitions

Parkinsonism subtypes with fewer than 500 subjects during the study period were excluded from the analysis. The three study outcomes of interest were PD, VP, and DIP. The R package CodelistGenerator was used to identify SNOMED CT diagnostic codes relevant to each outcome. As UK primary care records historically used Read codes, these were mapped to SNOMED CT concepts for the purposes of this study. These resulting computable phenotypes were then reviewed by clinicians with expertise in primary care and neurology based on clinical descriptions provided beforehand. These phenotypes were further refined using the R package CohortDiagnostics to assess their plausibility within the database. These diagnostics assessed whether key characteristics of identified cases—including age distributions, sex composition, comorbidities, and medication use—were consistent with established epidemiological patterns reported in the literature. This process also included identifying additional relevant codes and removing those deemed irrelevant, based on feedback from clinicians.

### Statistical methods

#### Baseline characteristics

Baseline characteristics were summarized for patients with a diagnosis of PD, VP, and DIP, respectively, using the R package PatientProfiles. Median and interquartile range (IQR) were used for continuous variables whereas counts and percentages were used for categorical variables. The baseline characteristics included age, sex, conditions any time prior and medications within 1 year prior to the first onset of each outcome.

#### Annual crude incidence rates

For each year of the study period, a crude incidence rate was calculated for each outcome. For each incidence calculation, an individual can contribute at most once. This means that only the first record of PD, VP, and DIP within the study period will be considered as incident cases. The incidence was calculated as a fraction between the number of incident cases and time at risk in person-years contributed by the background population that year. Rates, with 95% confidence intervals (CIs), were then expressed as per 100 000 person-years.

#### Annual crude period prevalence

Annual period prevalence was calculated; the denominator was the number of people in the background population, the numerator was the number of cases of each outcome that year. Prevalence was expressed as percentages with 95% CIs calculated.

#### Stratification and age-standardization

All results were stratified by age and sex. The age bands for the stratification were in 10 years except the first (18–30) and the last age band (80+). In accordance with CPRD reporting guidelines, we do not report results with fewer than five cases.

Results overall and by sex were age-standardized using 2013 European Standardised Population (ESP2013) [10]. ESP2013 serves as a standard age distribution to account for the change in age structure and offers an age-standardized perspective on secular trends. The age groups in ESP2013 were merged and scaled so that the age groups were in 10-years except the first one (18–29).

All incidence and prevalence analyses were performed using R (version 4.2.3) and the R package IncidencePrevalence [11]. The analytical code for this study can be accessed via https://github.com/oxford-pharmacoepi/ParkinsonsIncidencePrevalence.

## Results

### Baseline characteristics

Out of a total of 17 054 819 people available in CPRD GOLD from 1 January 2007 to 31 December 2021, 9 604 592 were eligible for this study. There was a total of 20 006, 1126, and 809 patients with a diagnosis of PD, VP and DIP, respectively (Table 1). The SNOMED CT codelists used are in Supplementary Table S1, and the CONSORT diagrams can be found in Supplementary Figs S1–S3.

**Table 1.** Baseline characteristics of patients with a diagnosis of PD, VP, and DIP between 2007 and 2021

Characteristic	PD	VP	DIP
Number of patients	20006	1129	809
Sex: Female, *n* (%)	7793 (39)	394 (39)	450 (55.6)
Age: Median (IQR)	75 (68–81)	80 (75–84)	71 (63–80)
Prior observation (IQR)	3930 (2318–5608)	4793 (3268–6131)	3783 (2119–5501)
Age bands: *n* (%)			
18–30	12 (0.1)	0 (0)	9 (1.1)
31–40	56 (0.3)	0 (0)	9 (1.1)
41–50	356 (1.8)	<5	33 (4.1)
51–60	1473 (7.4)	15 (1.3)	102 (12.6)
61–70	4412 (22.1)	107 (9.5)	225 (27.8)
71–80	8083 (40.4)	478 (42.3)	245 (30.3)
81–90	5146 (25.7)	477 (42.2)	170 (21)
91–100	467 (2.3)	50 (4.4)	15 (1.9)
>100	<5	0 (0)	<5
General conditions (any time prior): *n* (%)			
Pneumonia	558 (2.8)	62 (5.5)	42 (5.2)
Gastroesophageal reflux disease	666 (3.3)	58 (5.1)	25 (3.1)
CKD	3507 (17.5)	329 (29.1)	181 (22.4)
Stroke	841 (4.2)	189 (16.7)	59 (7.3)
Anxiety	3392 (17)	194 (17.2)	237 (29.3)
Dementia	1582 (7.9)	188 (16.7)	91 (11.2)
Cardiovascular disease^a^	6911 (34.5)	724 (64.1)	274 (33.9)
Hyperlipidaemia	2187 (10.9)	137 (12.1)	99 (12.2)
Diabetes	2346 (11.7)	226 (20)	130 (16.1)
Myocardial infarction	683 (3.4)	87 (7.7)	24 (3)
Venous thromboembolism	972 (4.9)	75 (6.6)	43 (5.3)
Malignant neoplastic disease	2754 (13.8)	224 (19.8)	100 (12.4)
Chronic obstructive pulmonary disease	1018 (5.1)	101 (8.9)	47 (5.8)
Heart failure	706 (3.5)	80 (7.1)	24 (3)
Osteoporosis	1218 (6.1)	120 (10.6)	46 (5.7)
Asthma	1628 (8.1)	96 (8.5)	69 (8.5)
Depressive disorder	3094 (15.5)	228 (20.2)	273 (33.7)
Hypertension	5902 (29.5)	434 (38.4)	198 (24.5)
Medications (1 year prior): *n* (%)			
Psycholeptics	4790 (23.9)	288 (25.5)	688 (85)
Diuretics	5520 (27.6)	436 (38.6)	222 (27.4)
Opioids	6049 (30.2)	391 (34.6)	274 (33.9)
Antibacterials systemic	8085 (40.4)	608 (53.9)	415 (51.3)
Systemic corticosteroids	1783 (8.9)	117 (10.4)	88 (10.9)
Beta blocking agents	5560 (27.8)	342 (30.3)	200 (24.7)
Drugs for obstructive airway disease	4049 (20.2)	257 (22.8)	167 (20.6)
Calcium channel blockers	4685 (23.4)	322 (28.5)	169 (20.9)
Anti-inflammatory antirheumatic	4682 (23.4)	212 (18.8)	191 (23.6)
RAS-acting agents	6918 (34.6)	524 (46.4)	221 (27.3)
Antiepileptics	2103 (10.5)	173 (15.3)	271 (33.5)
Drugs for acid related disorder	8006 (40)	566 (50.1)	387 (47.8)
Antidepressants	6365 (31.8)	446 (39.5)	468 (57.8)
Antithrombotics	3344 (16.7)	440 (39)	136 (16.8)
Drugs used in diabetes	2262 (11.3)	208 (18.4)	125 (15.5)
Lipid modifying agents	8810 (44)	711 (63)	390 (48.2)

DIP, drug-induced Parkinsonism; IQR, interquartile range; PD, Parkinson’s disease; VP, vascular Parkinsonism.

a Cardiovascular diseases included venous thrombosis, pulmonary embolism, peripheral vascular disease, ischemic heart disease, heart failure, heart disease, coronary arteriosclerosis, cerebrovascular disease, and atrial fibrillation.

PD and VP were more common in males (M: ∼60%) (Table 1) whereas DIP was more common in females (F: 55.6%). Those diagnosed with VP had the highest median age (80 years, IQR: 75–84) whereas the median age for DIP was the lowest (71 years, IQR: 63–80). Patients diagnosed with VP were more likely to have chronic kidney disease (CKD), dementia, stroke, and cardiovascular disease, and to be prescribed with diuretics, antithrombotics, Renin-angiotensin-system (RAS)-acting agents and lipid modifying agents within one year prior to the first onset compared to PD and DIP. Similarly, patients diagnosed with DIP were more likely to be diagnosed with anxiety and depressive disorder, and to be prescribed with antidepressants, antiepileptics and psycholeptics compared to PD and VP.

### Incidence

**Figure 1.**
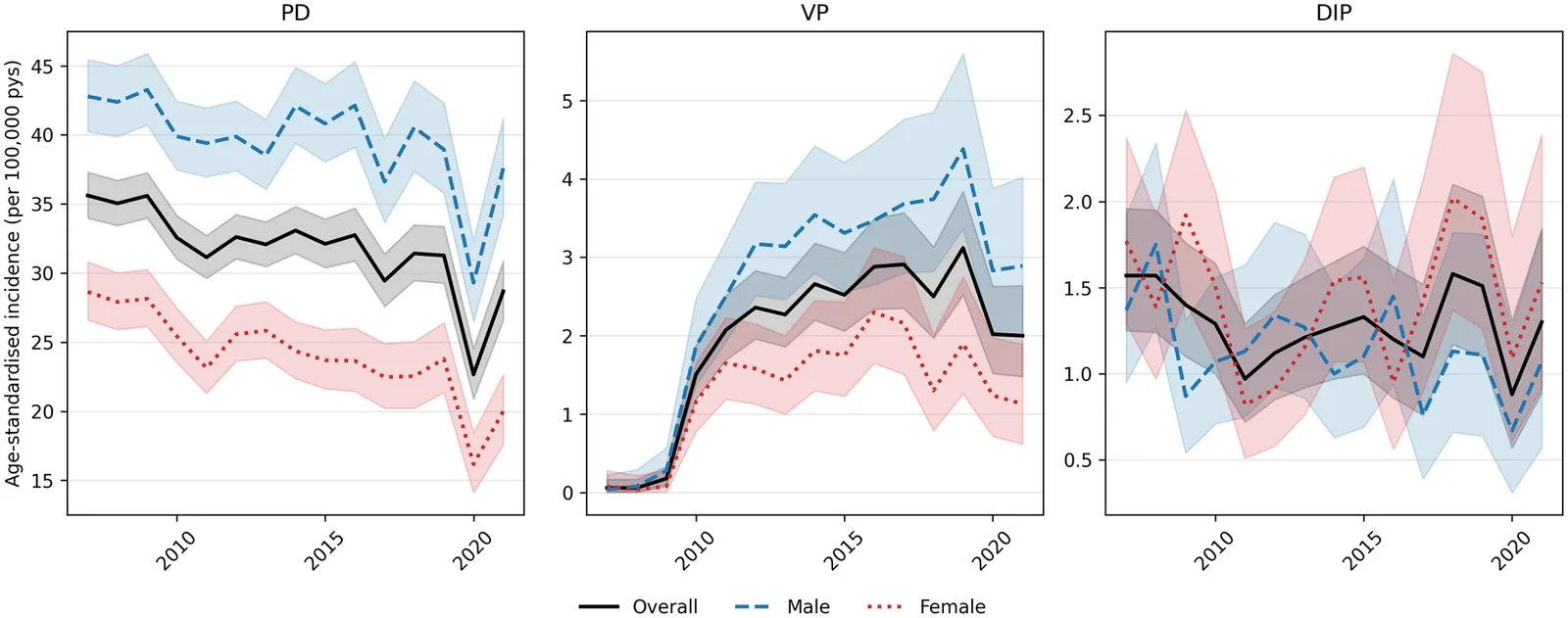
Age-standardized incidence rates of PD, VP, and DIP in CPRD GOLD between 2007 and 2021.

The annual incidence of PD decreased slightly from 2007 to 2019, with the age-standardized incidence rate being 35.61 per 100 000 person-years (pys) (95% CI: 33.97–37.30) and 31.27 per 100 000 pys (29.27–33.37) in 2007 and 2019, respectively (Fig. 1). The age-standardized incidence of VP increased from 0.18 (0.08–0.33) in 2009 to 2.00 in 2021 (1.48–2.64), peaking in 2019 with 3.12 per 100 000 pys (2.52–3.84). The incidence of DIP fluctuated between 0.8 and 1.6 per 100 000 pys. Crude results showed similar trends. When stratified by sex, both age-standardized and crude incidence of PD and VP was higher in males. The incidence of DIP for both sexes fluctuated but the incidence for females mostly remained slightly higher.

When stratifying by age, the crude incidence of Parkinsonism subtypes increased with age. For those aged over 70, the incidence of PD decreased whereas the incidence of VP increased. The rates for DIP were stable for all age bands considered. When stratifying by age and sex, the incidence of PD and VP remained higher for males across all age bands. No secular trends were observed for DIP.

All age-standardized and crude incidence results can be viewed and downloaded from an interactive Shiny web application (https://dpa-pde-oxford.shinyapps.io/ParkinsonismIncidencePrevalenceShiny/) or via Supplementary Table S2.

### Prevalence

**Figure 2.**
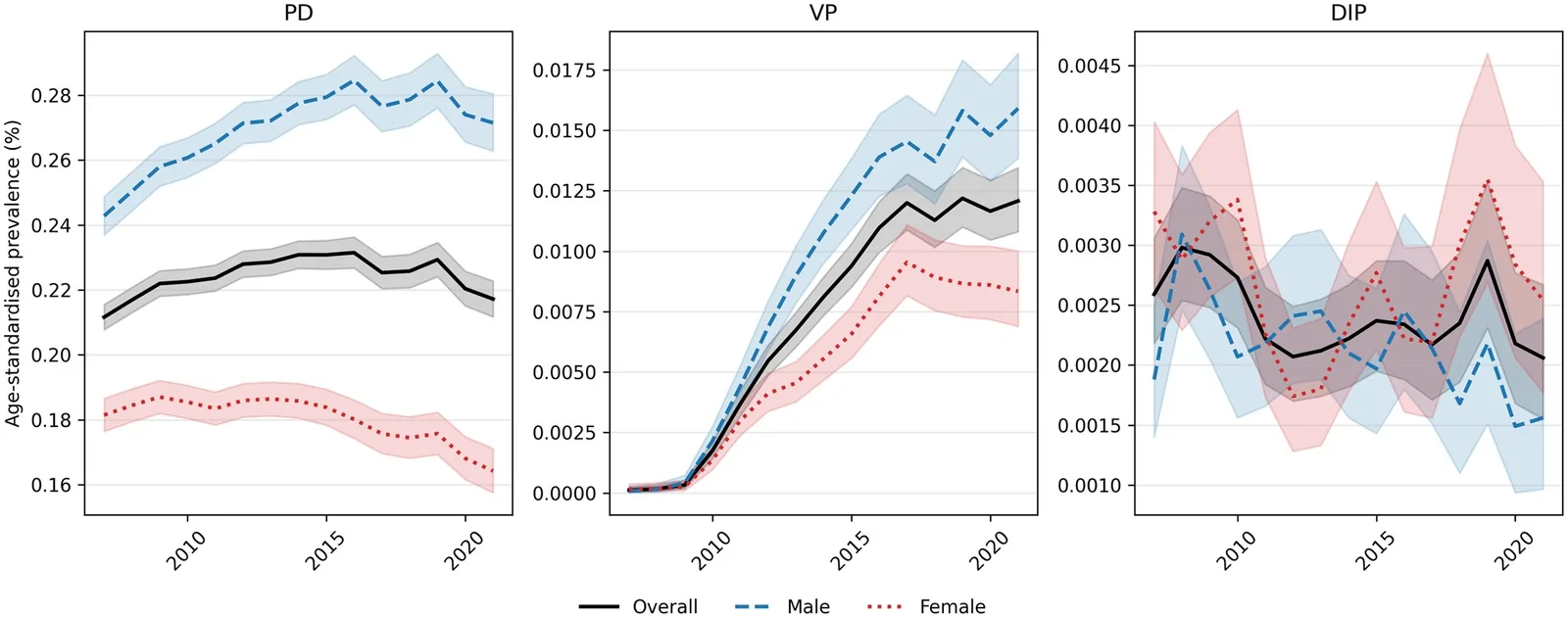
Age-standardized prevalence of PD, VP, and DIP in CPRD GOLD between 2007 and 2021.

From 2007, the age-standardized annual prevalence of PD gradually increased, reaching 0.23% (0.23%–0.24%) in 2016 (Fig. 2), before dipping from 2017 to 2018 and then peaking in 2019. The recording of VP increased from 2010, with prevalence remaining largely stable from 2017 onwards. The prevalence of DIP remained stable at 0.002%–0.003%. Age-standardized prevalence of PD was consistently higher in males, and the males showed increasing trends whereas females showed stable trends from 2007 to 2019. For VP, the number of recordings for both sexes increased from 2010 until 2019, where both remained stable thereafter. The prevalence of DIP for both sexes remained stable with females having slightly higher values than males during most of the years of the study period.

The crude prevalence of Parkinsonism subtypes increased with age, though such effect was not as strong for DIP. The prevalence of PD fluctuated slightly in many age groups considered, whereas for people aged over 70 the prevalence increased steadily. The prevalence of DIP remained stable. Stratifying by age and sex, PD prevalence decreased for females aged 61–70 but increased for males in the same group. For those over 80, PD prevalence decreased for both sexes. VP prevalence increased more in males than females over 80.

All age-standardized and crude prevalence results can be viewed and downloaded from an interactive Shiny web application (https://dpa-pde-oxford.shinyapps.io/ParkinsonismIncidencePrevalenceShiny/) or via Supplementary Table S3.

## Discussion

### Key results

This study provides a comprehensive assessment on the secular changes of the incidence and prevalence and characterization of those with a diagnosis of PD, VP and DIP in the UK. The incidence of PD has slightly decreased over time with the prevalence slightly increasing. The number of VP diagnoses increased progressively from 2010 to 2018 and remained stable thereafter. The incidence and prevalence of DIP remained stable over time. The incidence and prevalence of PD and VP were higher in males than females and such disparity increased with age.

### Demographic differences among individuals with Parkinsonism subtypes

In line with previous studies, the most frequently observed Parkinsonism subtype was PD [7, 12]. The second most common subtype was VP, contributing ∼5% of all subtype cases observed in this study, echoing The Rotterdam Study [12]. DIP contributed the least to total Parkinsonism cases in this work, which contrasts with some previous studies that state DIP is the second most prevalent subtype of Parkinsonism [3, 13]. This could be explained by the underdiagnosis of DIP as a medical condition [14] and misclassification of DIP [3]. Agreeing with literature, this study shows that males were twice as likely to develop PD and VP as females [15], DIP was slightly more common in females [13, 14, 16], and that the median age of onset of VP was later than PD [17]. However, there are conflicting results about the average age of DIP onset compared to PD; this work and other studies have shown lower average age of DIP onset compared to PD [18] while another study has shown the opposite [3]. Such contrast could be attributed partially to the large variation in age among people who use these causative medications. For example, although antipsychotics are used more frequently in older populations [19], studies have shown that there has been an increasing trend in antipsychotics prescriptions in children and adolescents [20].

Since VP is characterized by having a previous episode of ischemic cerebrovascular disease before showing Parkinson-like symptoms, it is unsurprising that these patients have a higher proportion of cardiovascular diseases compared to patients diagnosed with PD or DIP due to the overlapping risk factors and aetiologies between cardiovascular diseases and cerebrovascular diseases [21]. Although it is unsurprising that the number of stroke cases is higher in VP patients compared to other subtypes, the percentage of VP patients diagnosed with stroke was low (<20%). This could be explained by under-recording of stroke in primary healthcare [22]. Works have also demonstrated an association between cardiovascular disease and an increased risk of dementia [23], and that patients with CKD are more at risk of stroke [24], this may explain why the number of dementia and CKD cases are higher among VP patients.

Regarding DIP, these patients showed higher proportions of medications such as psycholeptics, antileptics, and antidepressants 1 year before DIP diagnosis. These medications are known dopamine receptor antagonists with strong evidence of inducing Parkinson-like symptoms [3, 14].

### Incidence and prevalence of PD

Prior studies in the UK reported higher incidence values than our study [6, 25]. For example, Okunoye *et al*. reported a crude incidence rate of 64.8 per 100 000 pys for PD in 2015 [6], while the same incidence rate was 32.3 per 100 000 pys in our study. This is due to different age distributions of the study population between these two studies. Our study includes those aged above 18 with Okunoye *et al*. including those aged above 50. For our study, the age-specific crude incidence rate for those aged above 50 ranged from ∼15 per 100 000 pys in those aged between 51 and 60 to ∼160 per 100 000 pys in those aged above 80. Our findings on secular trends show that the crude and age-standardized annual incidence of PD has decreased in the UK, which is concordant with literature [6, 25], and one potential reason for this slight decrease is the change of PD recordings in primary healthcare settings due to better recognition of other Parkinsonism subtypes as well as syndromes that share parkinsonian symptoms [25]. For instance, during 2018, there was a transition from Read Codes Version 2 (V2) to Clinical Terms Version 3 (CTV3) in 2018 [26], which may have influenced PD recordings. The more granular and detailed CTV3 coding system allows for better recognition of atypical Parkinsonism and parkinsonian syndromes such as DLB (DLB as a code did not exist in V2 of Read codes). Regarding prevalence, our results are in line with a Reference Report from Parkinson’s UK [27], also using CPRD GOLD, which estimated the crude prevalence of PD in 2015 at 0.27% in people aged over 18 compared to a prevalence of 0.23% from our work. Aligning with another study [28], our work shows that the prevalence of PD has increased over time. Since this pattern persists after age standardization, the increase could be driven by improved treatment and survival [28].

Concordant with existing literature, crude incidence and prevalence of PD are found to be higher in males [6, 18, 25, 29], and age-standardized results have shown the same pattern for this study. Factors that may have contributed to such a difference include genetics [30], a greater chance of developing traumatic head injury [31], and a greater exposure to adverse environmental factors due to working in industry sectors [32]. As expected, stratifying by age shows that incidence and prevalence of PD are higher in older age bands, and this can be explained by the nature of PD, which is characterized by the gradual and progressive presence of Lewy Bodies and loss of dopamine-producing neurons over time [33].

### Incidence and prevalence of VP

The increase of crude and age-standardized incidence and prevalence of VP could be attributed to a better recognition of this atypical disorder in the UK [25]. As a medical condition, VP has been heavily debated over the years [17]. It has been established as a new entity only in 2001 [34], and the first set of strict clinical criteria of VP been only established in 2004 [35]. Our study shows that the incidence and prevalence of VP are higher in males and one reason that could explain this finding is higher incidence of stroke in males [36], as stroke often precedes VP [17]. Moreover, it has also been shown that the severity of strokes tends to be higher in females, who are typically older and have higher mortality rates [37]. This may further reduce incidence and prevalence of VP for females; older age of stroke onset and higher mortality from stroke may mean a lesser chance of developing VP. This disparity between sexes increased with age, and it could be explained by males being more likely to experience multiple strokes [38].

### Incidence and prevalence of DIP

In contrast to PD and VP, this study finds that DIP has a higher incidence and prevalence among females, which agrees with previous literature [3, 39], and this remains true when we age standardize the results. The reasons for this are likely to be multifaceted. Females typically have higher body fat and lower body water percentages, which affects drug distribution and metabolism, and that hormonal differences such as oestrogen have been shown to influence drug metabolism [40]. Furthermore, females are also more likely to be prescribed medications that can induce DIP such as antipsychotics [19]. In contrast to Han *et al*. [39] where the incidence of DIP was decreasing, incidence and prevalence of DIP were both stable in this study. There are several reasons that can explain this difference including definition of DIP (the definition in Han *et al*. included the use of causative drugs) and population characteristics (the study in Han *et al*. was based in Asia).

Though studies have shown that the incidence or the prevalence of certain causative drugs such as antipsychotics have increased over the years [20], the incidence and prevalence of DIP has remained stable. A reason for this might be that these causative drugs are already very well-documented in literature [3], leading clinicians to carefully review patients’ medical histories and discontinue the use of these medications before they result in the full development of DIP.

### Strengths and limitations

This study has many strengths. Firstly, this study used CPRD GOLD, a large primary health care database covering GPs from England, Wales, Scotland and Northern Ireland, and thus may improve generalizability of the results. However, one may need to consider the change of the size of the database over time when interpreting the results. For example, the number of people in the database gradually decreased after 2011, likely due to GP practices in England moving to another clinical information system not captured in CPRD GOLD. Another strength of this study is that we have analysed the trends of the incidence and prevalence in each age and sex strata. In contrast to other studies [7], the inclusion of these stratified results can be useful in better informing which groups of people require more attention in terms of prevention.

One limitation could be the misclassification of these subtypes. Previous studies suggest that VP and DIP cases may be misclassified as PD by healthcare professionals [3, 14, 17]. Future research exploring stricter definitions for these subtypes is strongly recommended. For example, the inclusion of causative drugs prior the onset of DIP could be considered when defining DIP [39]. Another limitation is the use of the dataset. As CPRD GOLD is a primary care database, diagnoses made in secondary or specialist care are captured only if recorded by the GP. Consequently, some diagnoses may be under-recorded or recorded with delay, which could lead to underestimation of incidence and affect the timing of recorded diagnoses. One further limitation of this study relates to the requirement of at least 1 year of prior observation in CPRD GOLD before contributing to the denominator and time at risk. While longer disease-free lookback periods can further reduce misclassification of prevalent cases as incident, extending the lookback substantially reduces the eligible population and may bias the study population towards individuals with longer GP registration histories.

## Conclusion

Though the incidence of PD has been decreasing, the prevalence has been increasing, potentially suggesting improved survival and care. Both incidence and prevalence of VP have been steadily increasing which may suggest that there has been a change in how VP is diagnosed in the UK, and potentially a call for screening program for people who experience ischemic cerebrovascular diseases. The incidence and prevalence of DIP have been stable throughout the study period. As the population in the UK has been aging, Parkinsonism subtypes pose an ever-increasing burden.

## Supplementary Material

ckag031_Supplementary_Data

## Data Availability

This study is based in part on data from the CPRD obtained under license from the UK Medicines and Healthcare Products Regulatory Agency. The data is provided by patients and collected by the NHS as part of their care and support. The interpretation and conclusions obtained in this study are those of the authors alone. Patient level data used in this study was obtained through an approved application to the CPRD (Protocol number: 22_002351) and is only available following an approval process to safeguard the confidentiality of patient data. Details on how to apply for data access can be found at https://cprd.com/data-access. Key points From 2007 to 2021, the incidence of PD in the UK slightly decreased, while the prevalence increased a little. Both measures were higher in men. During the same period, the incidence and prevalence of Vascular Parkinsonism increased, again with higher rates in men. For DIP, the incidence and prevalence fluctuated over time and were slightly higher in females. The incidence and prevalence of all three conditions increased with age.

## Supplementary data

Conflict of interest: Financial disclosure related to research covered in this article: Professor Daniel Prieto-Alhambra research group has received research fundings from the European Medicines Agency and Innovative Medicines Initiative; and grants from Amgen, Chiesi-Taylor, Gilead, Lilly, Janssen, Novartis, and UCB Biopharma; and consultancy or speaker fees (paid to the University) from UCB Biopharma. Moreover, Janssen has funded or supported training programs organized by D.P.A’s department. All other authors declare no conflicts of interest.

Full financial disclosure for the previous 12 months: For the past 12 months, all coauthors except L.M.P are employed by the University of Oxford. A.J. has received grants from Data Analysis and Real World Interrogation Network (DARWIN EU^®^) [EMA] and Federated Analytics Initiative for Regulatory Science in the UK: FAIRS-UK [Innovate UK]. N.L.B. has participated in consultancies for Sleep Universal Limited, Theramex, and Hoffmann-Roche. D.P.A’s financial closure can be found in the paragraph above; moreover, D.P.A sits on the Board of the EHDEN Foundation. L.M.P. has received personal fees from Lilly and Esteve for educational activities, in addition, travel expenses for a conference have been covered by Esteve.
