# Supplementary figures and images for: Secular trends of incidence and prevalence of Parkinsonism subtypes: a cohort study in the United Kingdom
Source: Eur J Public Health. 2026 Apr 7;36(2):ckag031. doi: 10.1093/eurpub/ckag031 (PMC13064495; doi:10.1093/eurpub/ckag031)

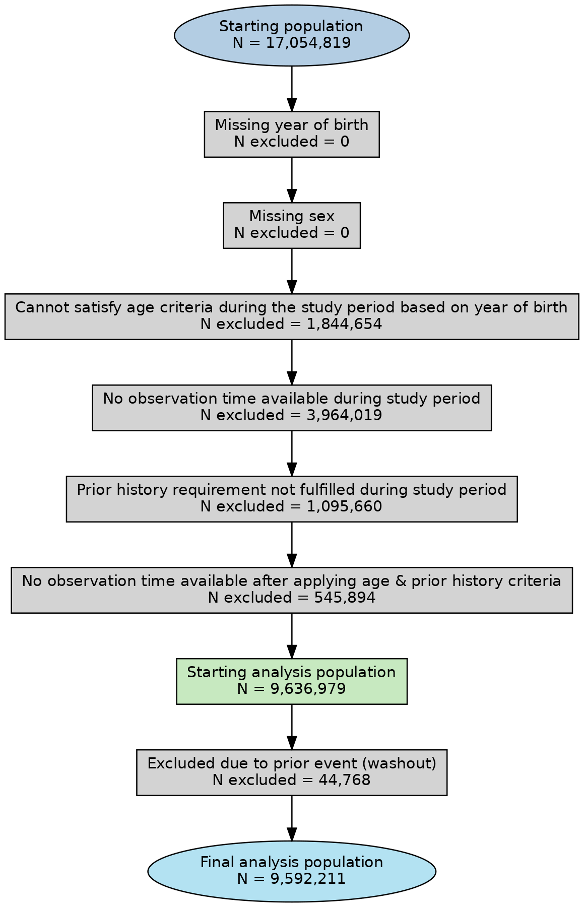

Supplement: ckag031_Supplementary_Data [file ckag031_supplementary_data.zip › ejph-2025-09-om-0805-File005.tiff]

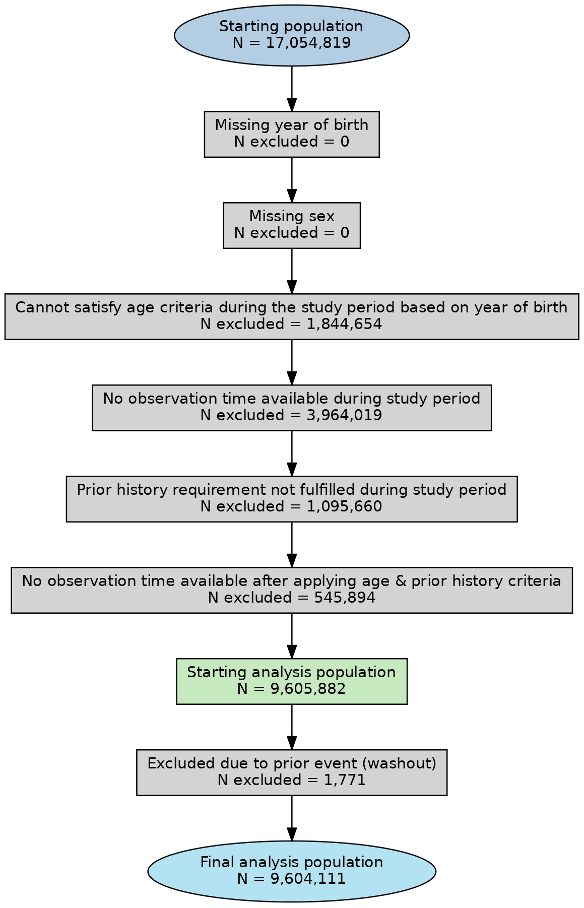

Supplement: ckag031_Supplementary_Data [file ckag031_supplementary_data.zip › ejph-2025-09-om-0805-File007.tiff]
